# TRENDY: gene regulatory network inference enhanced by transformer

**DOI:** 10.1093/bioinformatics/btaf314

**Published:** 2025-05-23

**Authors:** Xueying Tian, Yash Patel, Yue Wang

**Affiliations:** School of Information, University of California, Berkeley, CA 94720, United States; Department of Mathematics, University of Miami, Coral Gables, FL 33146, United States; Irving Institute for Cancer Dynamics and Department of Statistics, Columbia University, New York, NY 10027, United States

## Abstract

**Motivation:**

Gene regulatory networks (GRNs) play a crucial role in the control of cellular functions. Numerous methods have been developed to infer GRNs from gene expression data, including mechanism-based approaches, information-based approaches, and more recent deep learning techniques, the last of which often overlook the underlying gene expression mechanisms.

**Results:**

In this work, we introduce TRENDY, a novel GRN inference method that integrates transformer models to enhance the mechanism-based WENDY approach. Through testing on both simulated and experimental datasets, TRENDY demonstrates superior performance compared to existing methods. Furthermore, we apply this transformer-based approach to three additional inference methods, showcasing its broad potential to enhance GRN inference.

**Availability and implementation:**

Code and data files are available at https://github.com/YueWangMathbio/TRENDY, with DOI: 10.6084/m9.figshare.28236074.

## 1 Introduction

The expression of genes can be regulated by other genes. We can construct a directed graph, where vertices are genes, and edges are regulatory relationships. This graph is called a gene regulatory network (GRN). GRNs are essential to understanding the mechanisms governing cellular processes, including how cells respond to various stimuli, differentiate, and maintain homeostasis (Wang 2020, Wang *et al.* 2022). Understanding the GRN structure is important for developmental biology (Myasnikova and Spirov 2020, Wang *et al.* 2020, Cheng *et al.* 2022, 2024, Sha *et al.* 2024), disease research (Arshad *et al.* 2016, Angelini *et al.* 2022, Cheng *et al.* 2023, McDonald *et al.* 2023) and even studying macroscopic behavior (Li *et al.* 2021, Vijayan *et al.* 2022, Axelrod *et al.* 2023).

It is difficult to directly determine the GRN structure with experiments. Instead, researchers develop methods that infer the GRN structure through gene expression data. Some methods, such as WENDY (Wang *et al.* 2024) and NonlinearODEs (Ma *et al.* 2020), are mechanism-based: they construct dynamical models for gene expression, and fit with expression data to determine the GRN (Burdziak *et al.* 2023, Wang *et al.* 2023). Some methods, like GENIE3 (Huynh-Thu *et al.* 2010) and SINCERITIES (Papili Gao *et al.* 2018), are information-based: they treat this as a feature selection problem and directly find genes that can be used to predict the level of the target gene (e.g., by linear regression). Then this predictability (information) for the target gene is assumed to imply regulation (Huynh-Thu and Geurts 2018, Zheng *et al.* 2019).

Recently, there are some deep learning-based methods that infer GRN with different neural network frameworks, such as convolutional neural networks (Nauta *et al.* 2019), recurrent neural networks (Kentzoglanakis and Poole 2012), variational autoencoders (Shu *et al.* 2021), graph neural networks (Feng *et al.* 2023, Mao *et al.* 2023), etc. Deep learning frameworks can perform well on many tasks. However, because of the large number of tunable parameters in a neural network, it is difficult to explain why it works. These deep learning-based GRN inference methods have similar problems: they generally apply popular neural networks as black boxes without integration with biological mechanisms, and thus lacking interpretability.

The transformer model is a deep learning architecture designed to handle sequential data (Vaswani *et al.* 2017). It can capture long-range dependencies and relationships (linear and nonlinear) through the self-attention mechanism. The encoder layer of the transformer model can process the input through a neural network, so that the output is close to the given target.

Some researchers have developed GRN inference methods based on the transformer model (Xu *et al.* 2023) or its variants (Shu *et al.* 2022). The STGRNS method (Xu *et al.* 2023) predicts the existence of regulation between two genes solely based on the expression levels of these two genes. Since other genes are not considered, this approach makes it difficult to distinguish between direct regulation ($G_{i}\to G_{j}$) and indirect regulation through a third gene ($G_{i}\to G_{k}\to G_{j}$). The GRN-transformer method (Shu *et al.* 2022) uses multiple inputs, including the GRN inferred by an information-based method, PIDC (Chan *et al.* 2017), but it also does not consider the biological dynamics of gene regulation.

To combine the powerful deep learning techniques and biological understanding of gene regulation, we propose a novel GRN inference method that applies transformer models to enhance a mechanism-based GRN inference method, WENDY. This new method, TRansformer-Enhanced weNDY, is abbreviated as TRENDY. WENDY method is based on a dynamical model for gene regulation, and an equation for the GRN and the covariance matrix of genes is solved to derive the GRN. In TRENDY, we first use a transformer model to construct a pseudo-covariance matrix that performs better in WENDY. Then we apply another transformer model that directly enhances the inferred GRN.

The idea for the second half of TRENDY can be used to enhance the inferred results by other GRN inference methods. We apply transformer models to three GRN inference methods, GENIE3, SINCERITIES, and NonlinearODEs, to obtain their transformer-enhanced versions, tGENIE3, tSINCERITIES, and tNonlinearODEs.

There have been some research papers that study the idea of denoising networks (e.g. enhancing GRNs inferred by other methods), such as network deconvolution (ND) (Feizi *et al.* 2013), diffusion state distance (Cao *et al.* 2013), BRANE Cut (BC) (Pirayre *et al.* 2015), BRANE Clust (Pirayre *et al.* 2018), and network enhancement (Wang *et al.* 2018).

We test the four traditional methods (WENDY, GENIE3, SINCERITIES, and NonlinearODEs) with their enhanced versions (by transformer, ND, or BC) on two simulated data sets and two experimental data sets. All four transformer-enhanced methods outperform other methods, and TRENDY ranks the first among all methods.

In order to train a deep learning model, such as the transformer model used in this article, we need a sufficient amount of training data. Specifically, since we want the GRN inference method to work well in different situations, the training data should contain many different data sets that correspond to different GRNs. In reality, there are not many available experimental gene expression data sets with known GRNs. Therefore, we consider generating synthetic data sets.

To simulate new gene expression data, the most common approach is to assume that gene expression follows a certain mechanism, such as an ordinary differential equation (ODE) system (Hache *et al.* 2009, Kamimoto *et al.* 2023) or a stochastic differential equation (SDE) system (Schaffter *et al.* 2011, Dibaeinia and Sinha 2020). After determining the parameters of this system, one can simulate a discretized version of this system and obtain new data. Readers may refer to a review (Tripathi *et al.* 2017) for this mechanism approach. Another approach is to apply deep learning to learn from experimental gene expression data and generate new data that mimic the existing data. Such generative neural networks can be variational autoencoders (Shu *et al.* 2021) or generative adversarial networks (Marouf *et al.* 2020).

The advantage of the differential equation approach is that one can change the parameters to generate new data that correspond to another GRN, while the deep learning approach can only reproduce existing data. The disadvantage of the differential equation approach is that the modeled mechanism might not be a perfect fit to reality. Therefore, the generated data might be questionable, especially those generated by an oversimplified model, such as a linear ODE system. Instead, the deep learning approach can generate new data that are indistinguishable from the experimental data.

In this article, we need to feed the transformer models with data from many different GRNs. Therefore, we adopt the mechanism approach with a frequently used nonlinear and stochastic system (Pinna *et al.* 2010, Papili Gao *et al.* 2018, Wang *et al.* 2024):

(1) $$dX_{j}(t)=V\left\{\beta \prod_{i=1}^{n}\left[1+{\left(A_{\text{true}}\right)}_{i,j}\frac{X_{i}(t)}{X_{i}(t)+1}\right]-\theta X_{j}(t)\right\}dt+\sigma X_{j}(t)dW_{j}(t).$$

The matrix $A_{\text{true}}$ is a randomly generated ground truth GRN, where ${\left(A_{\text{true}}\right)}_{i,j}>0/=0/<0$ means that gene *i* has positive/no/negative regulatory effects on gene *j*. $X_{i}(t)$ is the expression level of gene *i* at time *t*, and $W_{j}(t)$ is a standard Brownian motion. For different genes, the corresponding Brownian motions are independent. The same as in previous papers (Pinna *et al.* 2010, Papili Gao *et al.* 2018, Wang *et al.* 2024), the parameter values are V=30, β=1, θ=0.2, and σ=0.1. Our goal is to infer $A_{\text{true}}$ from $X_{j}(t)$.

Most deep learning-based GRN inference methods are trained on experimental data sets, whose amount is very limited. Therefore, such methods need to carefully choose the neural network structure and the training procedure under this limitation. In this article, we consider a scenario in which the training data set is sufficiently large, and study how deep learning can achieve the best performance without data limitation.

## 2 Results

### 2.1 TRENDY method

WENDY method (Wang *et al.* 2024) uses single-cell gene expression data measured at two time points, each of size m×n (mRNA counts of *n* genes for *m* cells). Here we do not know how cells at different time points correspond to each other, since the measurement of single-cell level gene expression needs to kill the cells (Wang and Wang 2022). For data at two time points, the cell numbers *m* can be different.

For gene expression data at time points 0 and *t*, WENDY applies graphical lasso (Friedman *et al.* 2008) to calculate the covariance matrices for different genes, $K_{0}$ and $K_{t}$, each of size n×n. We can construct a general SDE system like Equation (1) to model the dynamics of gene regulation. After linearization of this system, one can obtain an approximated relation for $K_{0},K_{t}$, and the ground truth GRN $A_{\text{true}}$, also of size n×n:

(2) $$K_{t}=(I+tA_{\text{true}}^{T})K_{0}(I+tA_{\text{true}})+D+E.$$

Here *I* is an n×n identity matrix, *D* is an unknown n×n diagonal matrix, and *E* is the unknown error introduced by linearization. Then WENDY solves the GRN matrix *A* as a non-convex optimization problem:

(3) $$\arg\underset{A}{\min}f(A):=\frac{1}{2}\sum_{i\neq j}\{[K_{t}-(I+tA^{T})K_{0}(I+tA){]}_{i,j}{\}}^{2}.$$

Due to the unknown diagonal matrix *D*, this summation does not count elements with i=j. The result of this optimization problem is denoted as

$$A_{0}=\text{WENDY}(K_{0},K_{t}).$$

See Section 1, available as supplementary data at *Bioinformatics* online for details of WENDY.

If WENDY works perfectly, then

$$K_{t}=(I+tA_{0}^{T})K_{0}(I+tA_{0})+D,$$

meaning that $\left(I+tA_{\text{true}}^{T})K_{0}(I+tA_{\text{true}}\right)$ and $\left(I+tA_{0}^{T})K_{0}(I+tA_{0}\right)$ differ by *E*, the error introduced by the linearization, and $A_{0}$ may not match $A_{\text{true}}$ accurately. If we want to derive a more accurate and complicated equation of $K_{0}$, $K_{t}$, and $A_{\text{true}}$ than Equation (2), this equation might be numerically unstable to solve. Therefore, we need to find another way to improve WENDY.

Define

(4) $$K_{t}^{*}=(I+tA_{\text{true}}^{T})K_{0}(I+tA_{\text{true}}).$$

If we replace $K_{t}$ by $K_{t}^{*}$ in Equation (3) and solve *A*, then this inferred *A* should be very close to $A_{\text{true}}$. However, in practice, we only have $K_{0}$ and $K_{t}$, but not $K_{t}^{*}$, since $A_{\text{true}}$ is unknown.

The core idea is to train a transformer model TE(k=1) with input $K_{t}$, so that the output $K_{t}^{\prime }$ is close to $K_{t}^{*}$. Then we can calculate

$$A_{1}=\text{WENDY}(K_{0},K_{t}^{\prime }),$$

which is closer to $A_{\text{true}}$ than $A_{0}$. We can further improve $A_{1}$ using another transformer model TE(k=3), along with the information in $K_{0},K_{t}$, so that the output $A_{2}$ is even closer to $A_{\text{true}}$. These two transformer models will be discussed in the next subsection.

The actual $K_{t}$ is a complicated nonlinear function of $A_{\text{true}}$ and $K_{0}$. Thus $K_{t}^{*}$, also a function of $A_{\text{true}}$ and $K_{0}$, can be roughly learned by the TE(k=1) model as a function of $K_{t}$. Given accurate $K_{0}$ and $K_{t}^{*}$, we still cannot uniquely determine $A_{\text{true}}$, and the numerical solver for Equation (3) determines which $A_{1}$ is chosen. Therefore, the TE(k=3) model can partially learn the nonlinear behavior of the solver and output a better $A_{2}$ from $A_{1}$.

**Figure 1.**
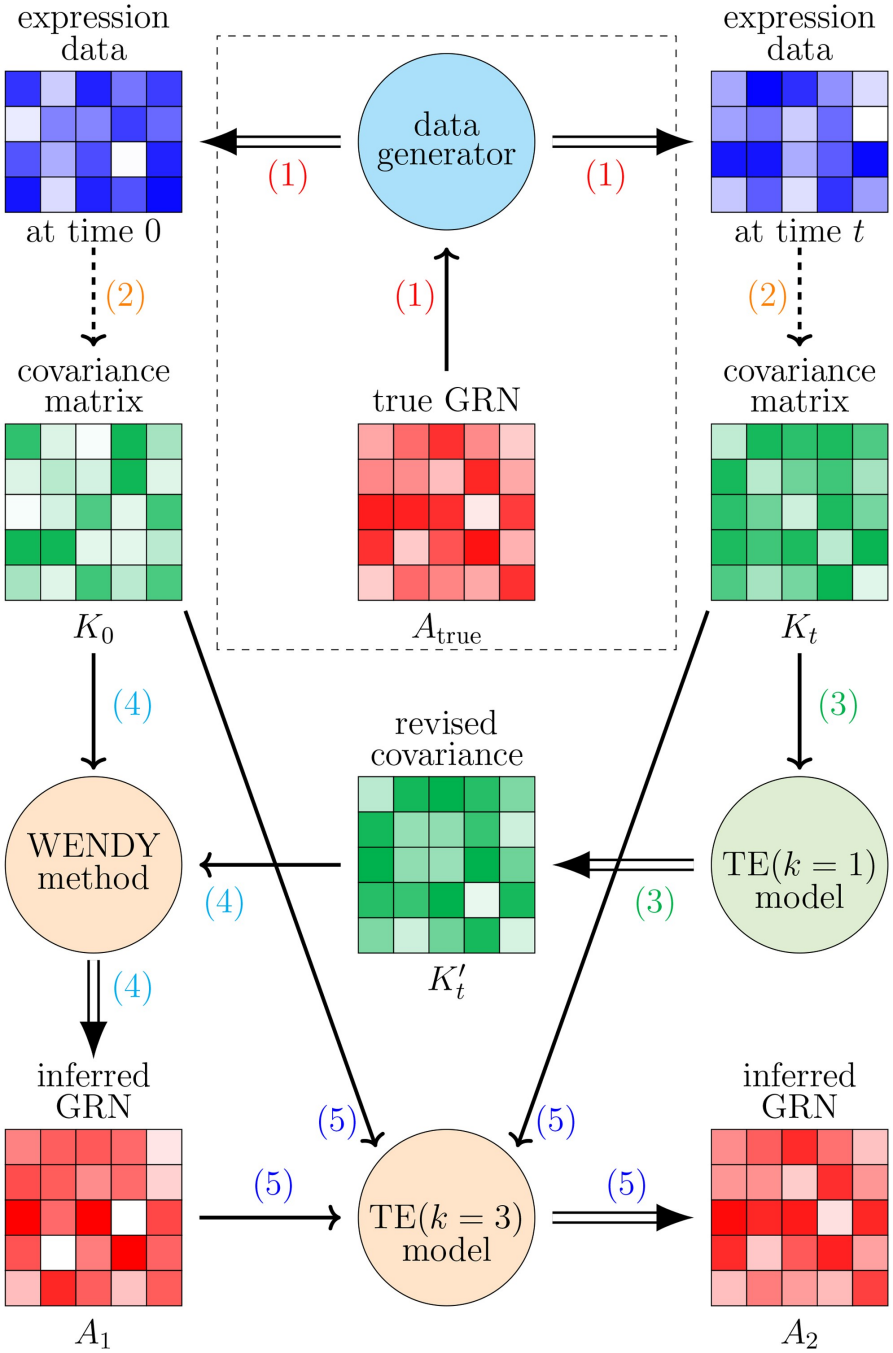
Workflow of training and testing the TRENDY method. Rectangles with grids are numerical matrices, where the color scale represents the numerical value. Circles are mechanisms that take in matrices and produce matrices. Single and double arrows represent inputs and outputs of each mechanism, and dashed arrows represent direct calculations. To apply TRENDY after training, the step in the dashed rectangle is omitted.

Figure 1, Algorithms 1 and 2 describe the training and testing workflow for TRENDY.

Algorithm 1 Training workflow of TRENDY method.

1. **Repeat** generating random $A_{\text{true}}$ and corresponding gene expression data

2. **Calculate** covariance matrices $K_{0}$ and $K_{t}$, and then calculate $K_{t}^{*}$ from $K_{0},K_{t},A_{\text{true}}$

3. **Train**  TE(k=1) model with input $K_{t}$ and target $K_{t}^{*}$  **Call** trained TE(k=1) model to calculate $K_{t}^{\prime }$ from $K_{t}$

4. **Call** WENDY to calculate $A_{1}$ from $K_{0},K_{t}^{\prime }$

5. **Train**  TE(k=3) model with input $A_{1},K_{0},K_{t}$ and target $A_{\text{true}}$

Algorithm 2 Testing workflow of TRENDY method.

1. **Input**: gene expression data at two time points

2. **Calculate** covariance matrices $K_{0}$ and $K_{t}$

3. **Call** trained TE(k=1) model to calculate $K_{t}^{\prime }$ from $K_{t}$

4. **Call** WENDY to calculate $A_{1}$ from $K_{0},K_{t}^{\prime }$

5. **Call** trained TE(k=3) model to calculate $A_{2}$ from $A_{1},K_{0},K_{t}$

6. **Output**: inferred GRN $A_{2}$

### 2.2 Transformer model TE(k)

We build a deep learning framework TE(k) that is based on transformer encoder layers, where *k* is the major hyperparameter that describes the number of input matrices. Besides, there are three hyperparameters that can be tuned: the model dimension *d*; the number of encoder layers *l*; the number of attention heads *h* of the encoder layer. We use this TE(k) model with different hyperparameters in TRENDY and other transformer-enhanced methods. See Algorithm 3 for the general structure of TE(k), along with the shape of data after each layer. Besides the standard transformer structure, the segment embedding layer tells the model to treat k>1 input matrices separately, and the 2-D positional encoding layer tells the model to remember that the input is originally two-dimensional. Notice that the same transformer encoder layer can handle inputs of different lengths. This means that the gene number *n* is not a predetermined hyperparameter, and we do not need to train a different model for each *n*. We will train the TE(k) model with n=10 genes and test on data sets with n=10/18/20 genes.

For the first half of the TRENDY method, the TE(k=1) model has k=1 input matrix $K_{t}$, d=64 model dimension, l=7 encoder layers, and h=4 attention heads. For the second half of the TRENDY method, the TE(k=3) model has k=3 input matrices of the same size, $A_{1},K_{0},K_{t}$, d=64 model dimension, l=7 encoder layers, and h=8 attention heads.

Algorithm 3 Structure of the TE(k) model. The shape of data after each layer is in the brackets.

1. **Input**: *k* matrices (*k* groups of n×n)

2. Linear embedding layer with dimension *d* (*k* groups of n×n×d)

3. Segment embedding layer (omitted when k=1) (*k* groups of n×n×d)

4. 2-D positional encoding layer (*k* groups of n×n×d)

5. Flattening and concatenation ($\left(n^{2})\times (dk\right)$)

6. *l* layers of transformer encoder ($\left(n^{2})\times (dk\right)$)

7. Linear embedding layer ($n^{2}\times 1$)

8. **Output**: reshaping into a matrix (n×n)

### 2.3 Other transformer-enhanced methods

The second half of the TRENDY method applies a transformer model to learn the highly nonlinear relation between $A_{1}$ (result of WENDY) and $A_{\text{true}}$. For other GRN inference methods, we can assume that the inferred GRN and $A_{\text{true}}$ have some nonlinear and possibly random relations, since these methods only consider and infer certain forms of regulations. Then we can try to enhance the inferred results by a transformer model.

GENIE3 (Huynh-Thu *et al.* 2010) works on single-cell expression data at one time point. It uses random forest to select genes that have predictability for the target gene, thus being information-based.

SINCERITIES (Papili Gao *et al.* 2018) works on single-cell expression data at multiple time points. It runs regression on the Kolmogorov–Smirnov distance between cumulative distribution functions of expression levels, also being information-based.

NonlinearODEs (Ma *et al.* 2020) works on bulk expression data at multiple time points. This method fits the data with a nonlinear differential equation system, which is thus mechanism-based.

For each of these three methods, we train a TE(k) model to enhance the inferred results, similar to the TE(k=3) model in the second half of TRENDY. Here each TE(k) model has d=64,l=7,h=8, and the training target is $A_{\text{true}}$.

For inferred GRN $A_{G}$ by GENIE3, we train a TE(k=2) model with $A_{G}$ and the covariance matrix *K* as input. Then we can use this trained model to calculate a more accurate $A_{G}^{\prime }$ from $A_{G}$ and *K*. This method is named tGENIE3.

For inferred GRN $A_{S}$ by SINCERITIES, we train a TE(k=1) model with $A_{S}$ as input, and name it tSINCERITIES.

For inferred GRN $A_{N}$ by NonlinearODEs, we train a TE(k=1) model with $A_{N}$ as input, and name it tNonlinearODEs.

### 2.4 Methods enhanced by ND and BC

Network deconvolution (ND) (Feizi *et al.* 2013) requires the input to be nonnegative and symmetric, so as its output. The idea is to assume that the input is a convolution of a simpler network, and solves this simpler network by deconvolution. BRANE Cut (BC) (Pirayre *et al.* 2015) performs well when we know that some genes are transcription factors and others are not. When we have no knowledge of transcription factors, BC degenerates to setting a threshold for whether one regulation exists. BC requires the input to be nonnegative and symmetric, and the output is a 0–1 matrix. We use ND and BC to enhance those four traditional methods, and name them as nWENDY, nGENIE3, nSINCERITIES, nNonlinearODEs, bWENDY, bGENIE3, bSINCERITIES, bNonlinearODEs.

### 2.5 Performance of different methods

Certain autoregulations can decrease the variance (Ramos and Reinitz 2019, Giovanini *et al.* 2020, Holehouse *et al.* 2020), and mRNA bursts can increase the gene expression variance (Paré *et al.* 2009, Bothma *et al.* 2014, Leyes Porello *et al.* 2023), which can be approximated by tuning the value of σ in Equation (1). SINC data set is generated by Equation (1) with σ=0.01/0.1/1. The synthetic DREAM4 data set (Marbach *et al.* 2012) is commonly used as a benchmark for GRN inference (Papili Gao *et al.* 2018, Wang *et al.* 2024). THP-1 data set measures monocytic THP-1 human myeloid leukemia cells (Kouno *et al.* 2013). hESC data set measures human embryonic stem cell-derived progenitor cells (Chu *et al.* 2016).

For these four data sets, two synthetic and two experimental, we test 16 methods: four traditional methods (WENDY, GENIE3, SINCERITIES, and NonlinearODEs), their transformer variants, ND variants, and BC variants. To compare the inferred GRN $A_{\text{pred}}$ and the ground truth GRN $A_{\text{true}}$, we adopt the common practice that calculates two area under curve (AUC) scores: AUROC and AUPRC (Wang *et al.* 2024). Both AUC scores are between 0 and 1, where 1 means perfect matching, and 0 means perfect mismatching. See Section 2, available as supplementary data at *Bioinformatics* online for details of these two scores.

**Figure 2.**
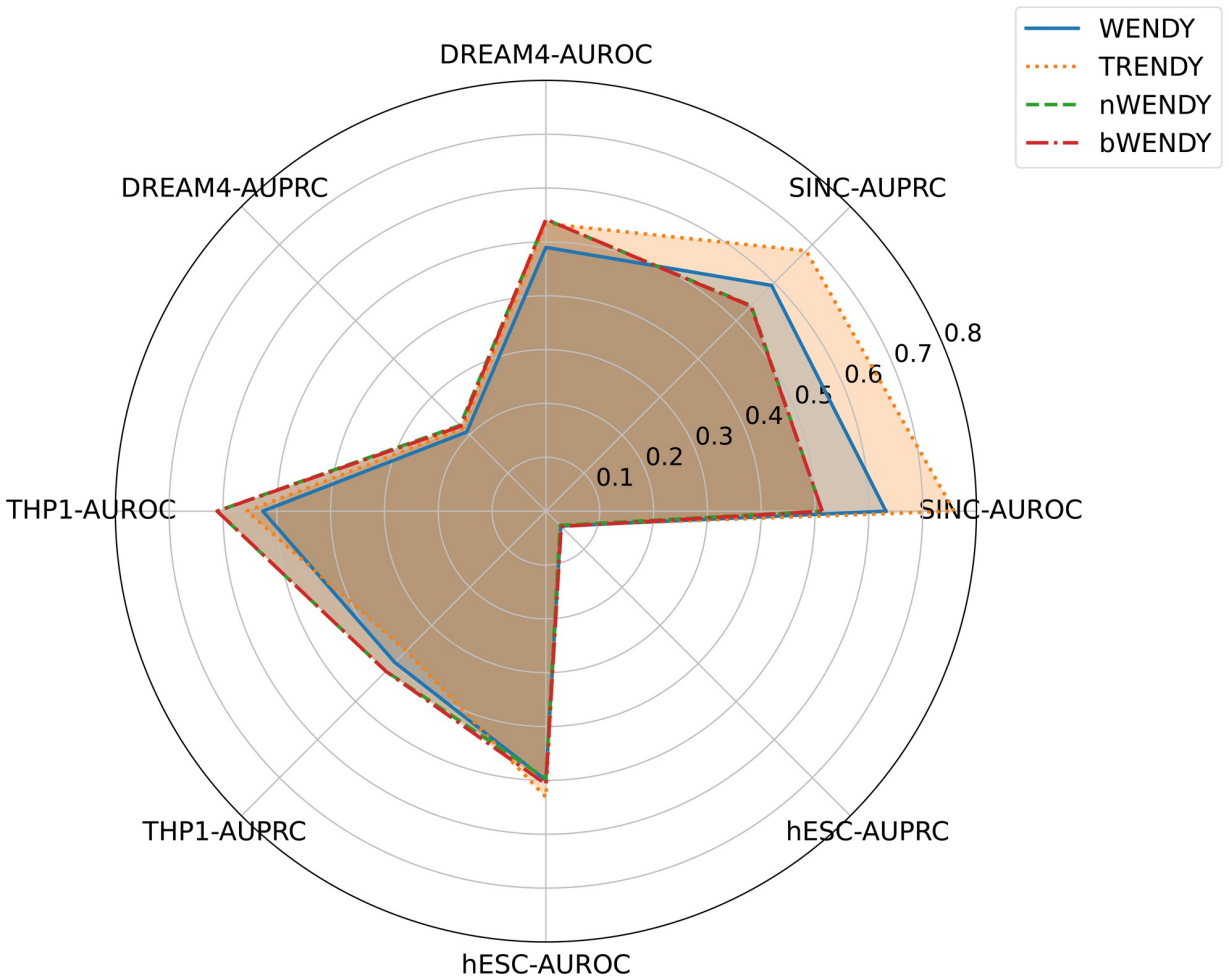
AUROC and AUPRC scores of WENDY and its variants on four datasets.

**Figure 3.**
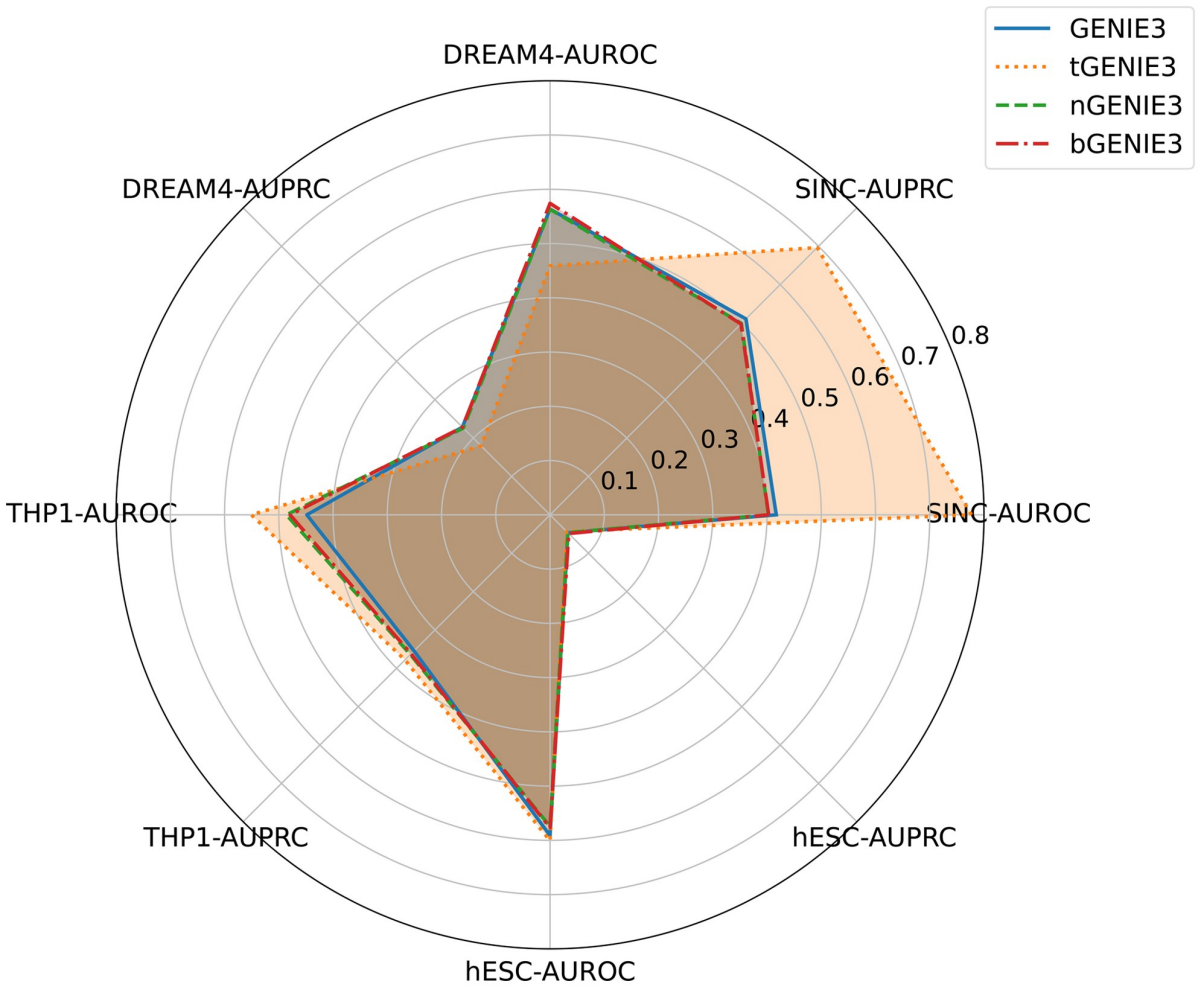
AUROC and AUPRC scores of GENIE3 and its variants on four datasets.

**Figure 4.**
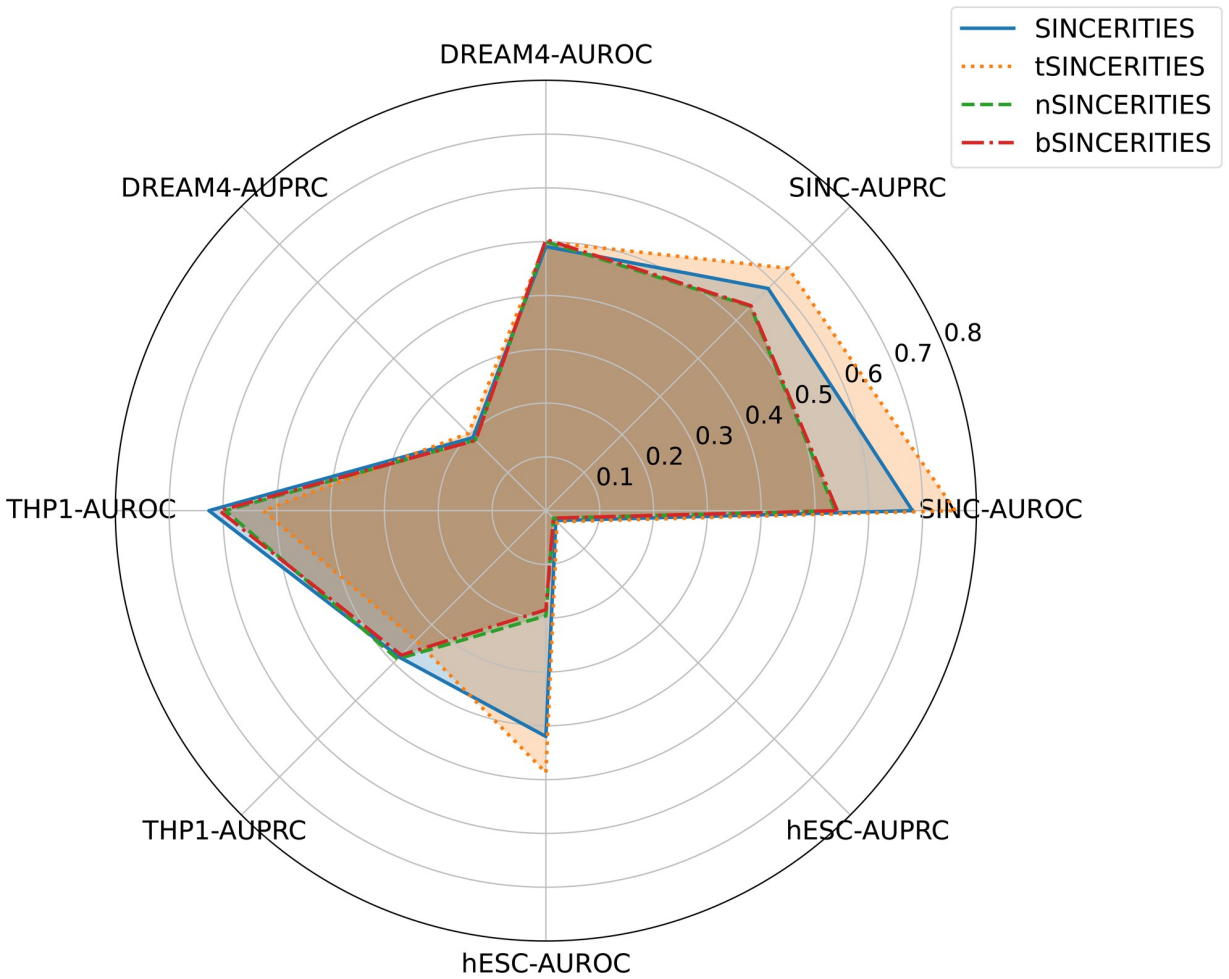
AUROC and AUPRC scores of SINCERITIES and its variants on four datasets.

**Figure 5.**
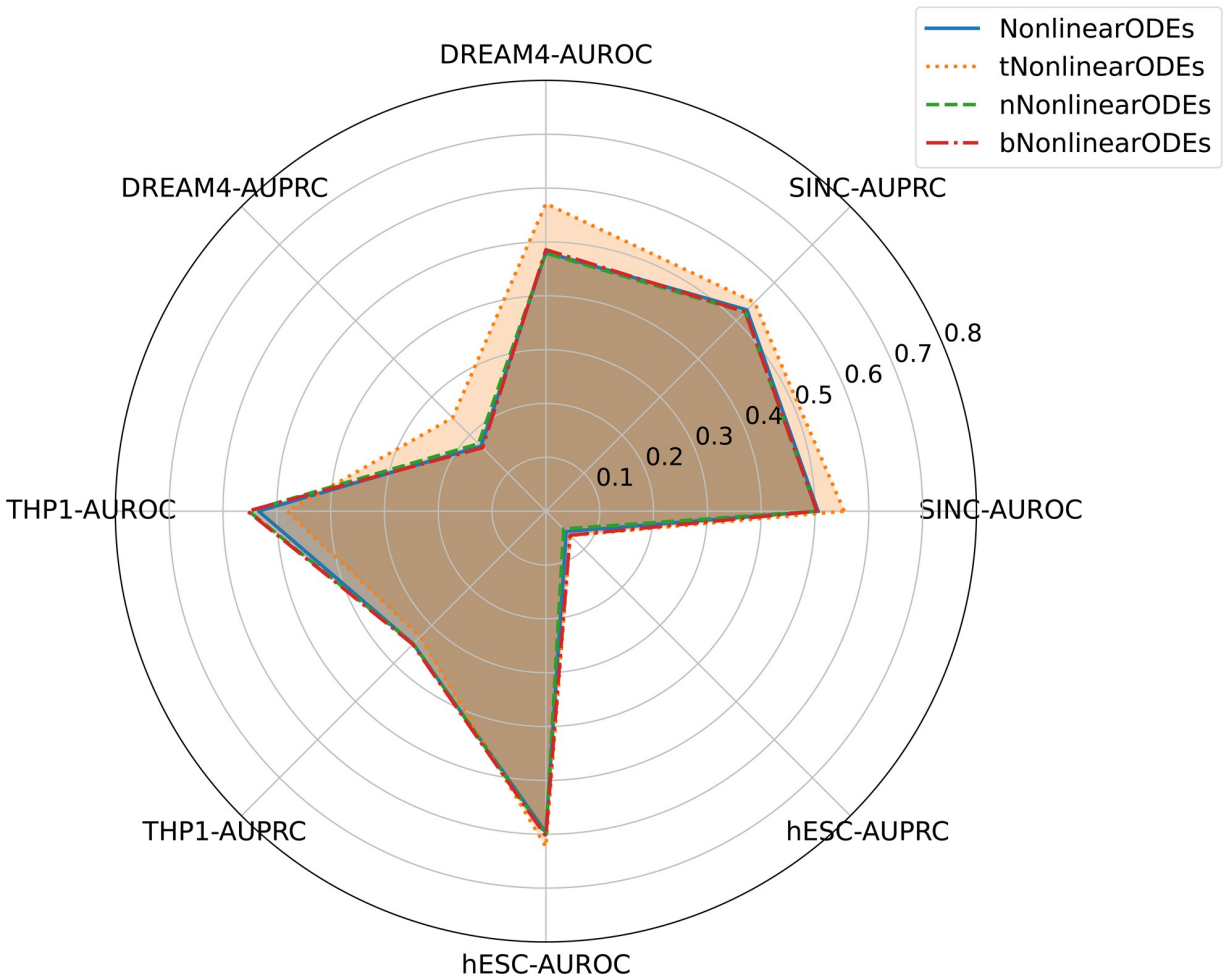
AUROC and AUPRC scores of NonlinearODEs and its variants on four datasets.

**Figure 6.**
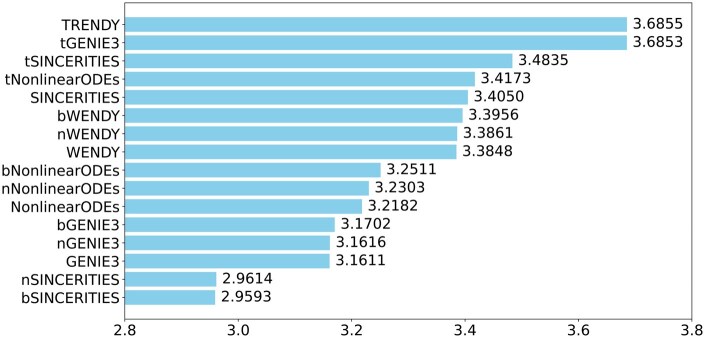
Total scores of all 16 methods, where the *x*-axis starts at 2.8.

See Figs 2–6 and Tables 1 and 2, available as supplementary data at *Bioinformatics* online for the performance of these methods. Although the training data only has n=10 genes, and the THP-1 and hESC data sets have n=20 and n=18 genes, the four transformer-enhanced methods (TRENDY, tGENIE3, tSINCERITIES, tNonlinearODEs) are better than all other methods. This means that our idea of enhancing GRN inference methods with TE(k) models works well universally. ND and BC generally do not enhance the original method significantly.

The core method of this article, TRENDY, ranks the first among all 16 methods (tGENIE3 is slightly inferior). Besides, the TRENDY method is better than the WENDY method on three data set, and has almost the same performance as WENDY on one data set (THP-1). The other three transformer-enhanced methods are worse than their non-transformer counterparts on at least one data set. This means that TRENDY’s performance is robust on different data sets. In sum, TRENDY has satisfactory performance among all methods tested.

All methods have very small AUPRC values on hESC data set. The reason is that the ground truth GRN $A_{\text{true}}$ has very few nonzero values (Fig. 3, available as supplementary data at *Bioinformatics* online).

## 3 Discussion

In this article, we present the TRENDY method, which uses the transformer model TE(k) to enhance the mechanism-based GRN inference method WENDY. TRENDY is tested against three other transformer-enhanced methods and their original forms, and ranks the first. This work explores the potential of deep learning methods in GRN inference when there are sufficient training data. Besides, TRENDY is developed on the biological dynamics of gene regulation, and it is more interpretable than other deep learning-based methods that treat GRN inference as a pure prediction task. Nevertheless, the transformer encoder is directly used as an oracle machine in TRENDY. A future direction is to modify the neural network structure to better represent the biological structure (Liu *et al.* 2020).

The essential difficulty of GRN inference is the lack of data with experimentally verified GRNs. Many deep learning models need significantly more data with known GRNs, and we have to generate new data. Since we do not fully understand the relation between the dynamics of gene expression and the ground truth GRN, it is difficult to evaluate the quality of generated data along with a random GRN. We also cannot guarantee that GRN inference methods that perform well on known experimental data sets can still be advantageous if there are many more experimental data with different GRNs.

We train the models with data of n=10 genes, and they work well on data with n=10/18/20 genes. If we want the models to work on data with many more genes, we need to train the models with corresponding data. However, when the gene number *n* is large, the number of different GRNs increases exponentially, and the number of training samples should also increase violently. The time cost for generating such data can be quite large. Even with the same amount of training samples, the training speed of the TE(k) model is proportional to $n^{4}$, since the input length of the transformer encoder layer is proportional to $n^{2}$, and it needs to calculate the attention scores between all pairs of input tokens. Therefore, scaling TRENDY for a much larger *n* is extremely time-consuming.

In this article, the training data are generated by Equation (1) that simulates the dynamics of mRNA count as a continuous-state process. This might be an oversimplification of gene regulation in reality, where the gene state and the protein count also should be considered (Holehouse *et al.* 2020). Besides, the experimental gene expression data often suffer from incomplete measurement, where many mRNAs are not recorded, and there are many missing values. Therefore, when we train on perfectly measured data and test on data with missing values, the performance is not guaranteed. We can manually add measurement errors and missing values to the training data to solve this problem.

In our testing, we find that adding covariance matrices in the transformer (TRENDY and tGENIE3) is beneficial, since the covariance matrices may contain extra information besides that contained in the inferred GRN.

TRENDY uses covariance matrices instead of raw gene expression data as input. One advantage is that calculating covariance matrices by graphical lasso regularizes the data. One disadvantage is that covariance only concerns second-order moment, while information contained in higher-order moments is abandoned.

## 4 Methods

### 4.1 Training data

To generate training data for our TRENDY method, we use Equation (1) to numerically simulate gene expression data with the Euler-Maruyama method (Kloeden *et al.* 1992) for n=10 genes. Every time we generate a random ground truth GRN $A_{\text{true}}$, where each element has probability 0.1/0.8/0.1 to be −1/0/1. Then we use Eq. 1 with this random $A_{\text{true}}$ to run the simulation from time 0.0 to time 1.0 with time step 0.01 to obtain one trajectory, where the initial state at time 0 is random. This is repeated 100 times to obtain 100 trajectories, similar to the situation where we measure the single-cell expression levels of *n* genes for 100 cells (Qian and Cheng 2020). We only record expression levels at time points 0.0,0.1,0.2,…,1.0. We repeat the simulation to obtain $1.01\times 10^{5}$ samples, where $10^{5}$ samples are for training, and $10^{3}$ samples are for validation (used in hyperparameter tuning and early stopping).

### 4.2 Loss function

We use AUROC and AUPRC to measure the difference between $A_{\text{true}}$ and the inferred $A_{\text{pred}}$. See Section 2, available as supplementary data at *Bioinformatics* online for details. Unfortunately, for fixed $A_{\text{true}}$ and arbitrary $A_{\text{pred}}$, AUROC and AUPRC can only take finitely many values, and are not continuous functions of $A_{\text{pred}}$. We cannot train a neural network with a non-differentiable loss function like AUROC or AUPRC. There have been some surrogate loss functions for AUROC or AUPRC (Qi *et al.* 2021, Yuan *et al.* 2023). We directly use the mean square error loss function, which also performs well. The loss for comparing two GRNs does not count the diagonal elements, since they represent the autoregulation that cannot be inferred by the methods in this article (Wang and He 2023).

### 4.3 Segment embedding

For TE(k) model with k>1 input matrices, after processing them with the linear embedding layer, we need to add segment embeddings to them, so that the model can differentiate between different input matrices (Devlin *et al*. 2019). We give the segment ID 0/1/2/… to the first/second/third/… group of processed inputs, and use an embedding layer to map each segment ID to a *d*-dimensional embedding vector. Repeat this embedding vector n×n times to obtain an (n×n×d) array, and add it to the (n×n×d) arrays after the linear embedding layer. In our numerical simulations, we find that this segment embedding layer slightly improves the performance.

### 4.4 Positional encoding

For the TE(k) model, the 2-dimensional positional encoding layer (Xu *et al.* 2023) adds an (n×n×d) array PE to each of the embedded input, so that the model knows that the input is two-dimensional. For *x* and *y* in 1,2,…,n and *j* in 1,2,…,d/4, PE is defined as

(5) $$\begin{array}{ll} & \text{PE}[x,y,2j-1]=\text{PE}[x,y,2j-1+d/2]\\ =\text{sin}[(y-1)\times 10^{-32(j-1)/d}],\\ & \text{PE}[x,y,2j]=\text{PE}[x,y,2j+d/2]\\ =\text{cos}[(y-1)\times 10^{-32(j-1)/d}],\\ & \text{PE}[x,y,2j-1+d/4]=\text{PE}[x,y,2j-1+3d/4]\\ =\text{sin}[(x-1)\times 10^{-32(j-1)/d}],\\ & \text{PE}[x,y,2j+d/4]=\text{PE}[x,y,2j+3d/4]\\ =\text{cos}[(x-1)\times 10^{-32(j-1)/d}]. \end{array}$$

In our numerical simulations, we find that this 2-D positional encoding is crucial for the TE(k) model to produce better results.

### 4.5 Training setting and cost

All TE(k) models used in this article are trained for 100 epochs. For each epoch, we evaluate the model performance on the validation data set. If the performance does not improve for 10 consecutive epochs, we stop training early. For all transformer encoder layers, the dropout rate is 0.1. The optimizer is Adam with learning rate 0.001.

Data generation and model training are conducted on a desktop computer with Intel i7-13700 CPU and NVIDIA RTX 4080 GPU. Data generation takes about one week with CPU parallelization. Training for each TE(k) model takes about two hours with GPU acceleration. After training, the time cost of applying TRENDY on a data set with ∼10 genes and ∼100 cells is under one second.

## Supplementary Material

btaf314_Supplementary_Data
