# Supplementary material for: TRENDY: gene regulatory network inference enhanced by transformer
Source: Bioinformatics. 2025 May 23;41(6):btaf314. doi: 10.1093/bioinformatics/btaf314 (PMC12133277; doi:10.1093/bioinformatics/btaf314)
Supplement: btaf314_Supplementary_Data [file btaf314_supplementary_data.pdf]

Supplementary Material of "TRENDY: Gene Regulatory Network Inference Enhanced by Transformer"

Xueying Tian, Yash Patel, and Yue Wang

S1 Details of WENDY method

We start with a general SDE for gene expression levels $X_j(t)$:

$$\frac{\mathrm{d}X_i(t)}{\mathrm{d}t} = f(X_1, \ldots, X_n) + c_{1,i} - c_{2,i}X_i + \text{noise}.$$

Here f describes the interactions of different genes; $c_{1,i}$ is the synthesis rate; $c_{2,i}$ is the degradation rate.

After some simplifications and linearizations, we have

$$\frac{\mathrm{d}X(t)}{\mathrm{d}t} = X(t)A + c + X(t) \odot \mathrm{d}\sigma W(t).$$

Here $X(t) = [X_1(t), \ldots, X_n(t)]$, A is the GRN, $W(t)$ is an n-dimensional standard Brownian motion, and $\odot$ is the entrywise (Hadamard) product.

Its approximated solution is

$$X(t) = X(0)(I + tA) + tc + X(0) \odot \epsilon(t),$$

where $\epsilon(t) = [\epsilon_1(t), \ldots, \epsilon_n(t)]$ is an n-dimensional normal random noise with 0 mean and diagonal covariance matrix.

Then we can calculate the covariance matrix

$$K(t) = \mathbb{E}\{[X(t)^{\mathrm{T}} - \mathbb{E}X(t)^{\mathrm{T}}][X(t) - \mathbb{E}X(t)]\},$$

and the final equation is

$$K(t) = (I + tA^{\mathrm{T}})K(0)(I + tA) + D + E,$$

where D is an unknown diagonal matrix that depends on the variance of ϵ, and E is the error introduced by linearizations.

WENDY method solves A from the last equation by the BFGS algorithm.

S2 Calculation of AUROC and AUPRC

In our setting, each edge in the true GRN can only take three values (labels): -1 (negative regulation), 0 (no regulation), and 1 (positive regulation). In the inferred GRN, each edge corresponds to a real number that can be mapped to those three labels. We need to evaluate the inferred GRN as a classification problem.

AUROC (Area Under the Receiver Operating Characteristic Curve) and AUPRC (Area Under the Precision-Recall Curve) are metrics for evaluating the performance of classification models (originally for binary classification). They are both between 0 and 1. If the case has perfect match, where the sorted predicted results and the sorted true labels have the same order, then AUROC and AUPRC are 1. If the order is fully reversed, AUROC and AUPRC are 0. Notice that only the order matters, and the predicted results do not need to match the true labels numerically. Although they have similar properties, AUROC is more valuable when different true labels are relatively balanced, and AUPRC is more informative with imbalanced labels. Therefore, we use both to evaluate the inferred GRN.

We use a toy example to illustrate the calculation of AUROC and AUPRC, given the ground truth GRN and the inferred GRN.

Assume that the ground truth GRN and the inferred GRN are

$$A_{\text{true}} = \begin{bmatrix} 0 & 0 & 0 & 1 & 1 \\ 1 & 1 & -1 & 0 & 0 \\ 0 & -1 & 1 & 0 & 1 \\ 1 & -1 & -1 & 1 & 0 \\ 0 & -1 & -1 & -1 & -1 \end{bmatrix},$$

and

$$A_{\text{pred}} = \begin{bmatrix} 0.45 & 0.15 & 0.30 & -0.94 & 0.24 \\ 0.02 & 0.09 & -0.28 & -0.84 & -0.61 \\ 0.90 & -0.18 & -0.07 & -0.89 & -0.92 \\ 0.43 & 0.08 & 0.02 & -0.51 & 0.49 \\ -0.66 & -0.62 & -0.08 & -0.43 & -0.51 \end{bmatrix}.$$

Since all GRN inference methods in the main text cannot handle autoregulation, we omit edges (i, i). For each edge (i, j) with $i \neq j$, we know the true label $A_{\text{true}}[i, j]$ and the predicted value $A_{\text{pred}}[i, j]$. Since the true label has three possibilities, but AUROC and AUPRC are originally defined for binary classification, we need to consider every two different labels.

Consider all edges (i, j) with $i \neq j$ and true label 0 or 1. List $(A_{\text{pred}}[i, j], A_{\text{true}}[i, j])$ and sort by $A_{\text{pred}}[i, j]$. Then we have (-0.94, 1), (-0.92, 1), (-0.89, 0), (-0.84, 0), (-0.66, 0), (-0.61, 0), (0.02, 1), (0.15, 0), (0.24, 1), (0.30, 0), (0.43, 1), (0.49, 0), (0.90, 0).

For a given threshold T, we can count
true positives (TP): $A_{\text{true}}[i, j] = 1$, $A_{\text{pred}}[i, j] > T$;
true negatives (TN): $A_{\text{true}}[i, j] = 0$, $A_{\text{pred}}[i, j] \leq T$;
false positives (FP): $A_{\text{true}}[i, j] = 0$, $A_{\text{pred}}[i, j] > T$;
false negatives (FN): $A_{\text{true}}[i, j] = 1$, $A_{\text{pred}}[i, j] \leq T$.

Then we can calculate
true positive rate (TPR): TP/(TP+FN);
false positive rate (FPR): FP/(FP+TN).

For instance, if the threshold to be 0, then
TP = 3, TN = 4, FP = 4, FN = 2, TPR = 0.6, FPR = 0.5.

We let the threshold T change from $\min(A_{\text{pred}})$ to $\max(A_{\text{pred}})$, and draw the corresponding points (FPR, TPR) in the square $[0, 1] \times [0, 1]$: (0.0, 0.0), (0.125, 0.0), (0.25, 0.0), (0.25, 0.2), (0.375, 0.2), (0.375, 0.4), (0.5, 0.4), (0.5, 0.6), (1.0, 0.6), (1.0, 1.0). Connect these points to obtain the receiver operating characteristic (ROC) curve, and AUROC is the area under this curve. See Fig. S1 for the illustration, where AUROC is 0.375.

For true labels -1 and 1, repeat this procedure, and the AUROC[-1,1] is 0.571. For true labels -1 and 0, repeat this procedure, and the AUROC[-1,0] is 0.518.

AUROC for two labels has an equivalent definition: in all pairs of edges (i, j) with $i \neq j$ and (p, q) with $p \neq q$, so that $A_{\text{true}}[i, j] < A_{\text{true}}[p, q]$, AUROC is the proportion that $A_{\text{pred}}[i, j] < A_{\text{pred}}[p, q]$.

Inspired by this definition, the AUROC for three labels should be the weighted average of three AUROC for two labels, where the weight is the number of pairs with different labels. In this example, there are 5 edges with true label 1, 8 edges with true label 0, and 7 edges with true label -1. Therefore, the final AUROC is

$$\frac{5 \times 8 \times \text{AUROC}[0, 1] + 5 \times 7 \times \text{AUROC}[-1, 1] + 8 \times 7 \times \text{AUROC}[-1, 0]}{5 \times 8 + 5 \times 7 + 8 \times 7} = 0.489.$$

To calculate AUPRC, just replace TPR by Precision=TP/(TP+FP), and replace FPR by Recall=TP/(TP+FN).

S3 Training and testing details

During training, for variants of WENDY, since they only need two time points, we fix the first time point to be 0.0, and the second time point can be $0.1, 0.2, \ldots, 1.0$. Therefore, each simulated sample corresponds to 10 pairs of K_0 and K_t. This means that the training set has 10^6 pairs of K_0 and K_t, where each t in $0.1, 0.2, \ldots, 1.0$ corresponds to 10^5 pairs. For variants of GENIE3, we consider each time point t in $0.1, 0.2, \ldots, 1.0$ and calculate the inferred GRN A_{G}. Then there are 10^6 pairs of A_{G} and K_t for training the TE($k = 2$) model. For variants of SINCERITIES and NonlinearODEs, we use the data

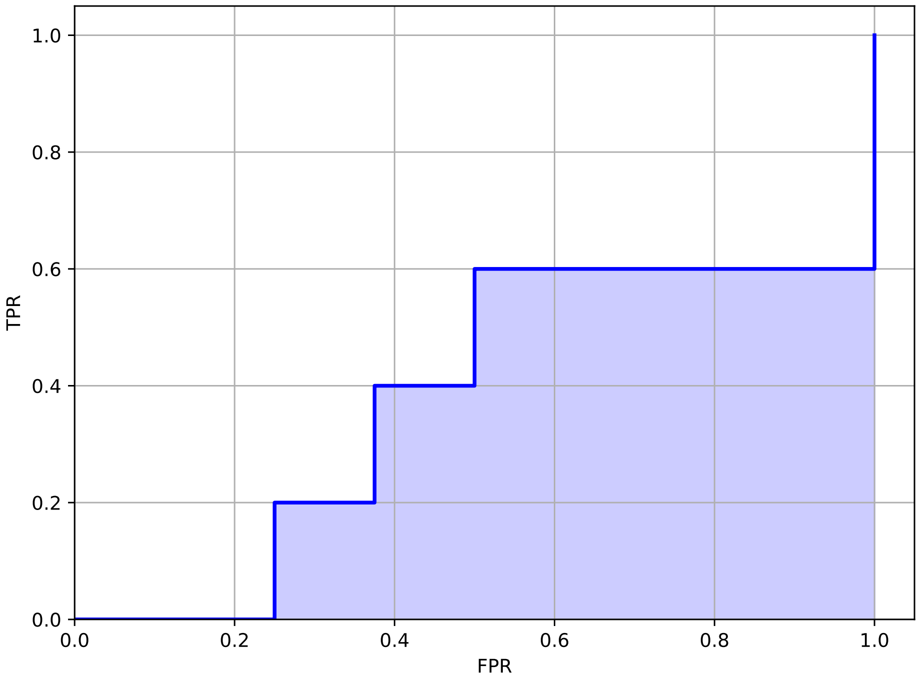

Figure S1: Example of ROC curve, where AUROC is the shaded area.

at all time points $0.0, 0.1, 0.2, \ldots, 1.0$ to infer the GRN A_S and A_N. Therefore, each method has 10^5 samples for training the TE($k = 1$) model.

For SINC data set, there are 1000 samples for each value of $\sigma = 0.01/0.1/1$, and each sample with a random A_{true} has 100 cells measured at time points $0.0, 0.1, \ldots, 1.0$. For WENDY and its variants, we set the first time point to 0.0 and vary the second time point from 0.1 to 1.0. For GENIE3 and its variants, we set the time point to any of $0.1, \ldots, 1.0$. For SINCERITIES, NonlinearODEs, and their variants, we use all time points.

For DREAM4 data set, we use the 10-gene time series data set. There are five A_{true}, each corresponding to the levels of 10 genes for five cells at 21 time points. For WENDY and its variants, we consider any pair of time points and take average. For GENIE3 and its variants, we consider any one time point and take average. For SINCERITIES, NonlinearODEs, and their variants, we use any consecutive 11 time points and take average.

For the THP-1 data set, there are eight time points, each with 120 cells measured. The same as in a previous paper [Wang et al., 2024], we consider the expression levels of 20 genes. See Fig. S2 for the ground truth GRN. For WENDY and its variants, we consider any pair of time points and take average. For GENIE3 and its variants, we consider any one time point and take average. For SINCERITIES, NonlinearODEs, and their variants, we use all eight time points.

For the hESC data set, there are six time points, each with 66–172 cells measured. The same as in a previous paper Wang et al. [2024], we consider the expression levels of 18 genes. See Fig. S3 for the ground truth GRN. For WENDY and its variants, we consider any pair of time points and take average. For GENIE3 and its variants, we consider any one time point and take average. For SINCERITIES, NonlinearODEs, and their variants, we use all six time points.

S4 Heat maps of true GRN for experimental data

See Fig. S2 for the heat map of the true GRN of THP-1 data set. Possible values are 1, -1, and 0, meaning positive regulation, negative regulation, and no regulation.

See Fig. S3 for the heat map of the true GRN of hESC data set. Possible values are 1 and 0, meaning

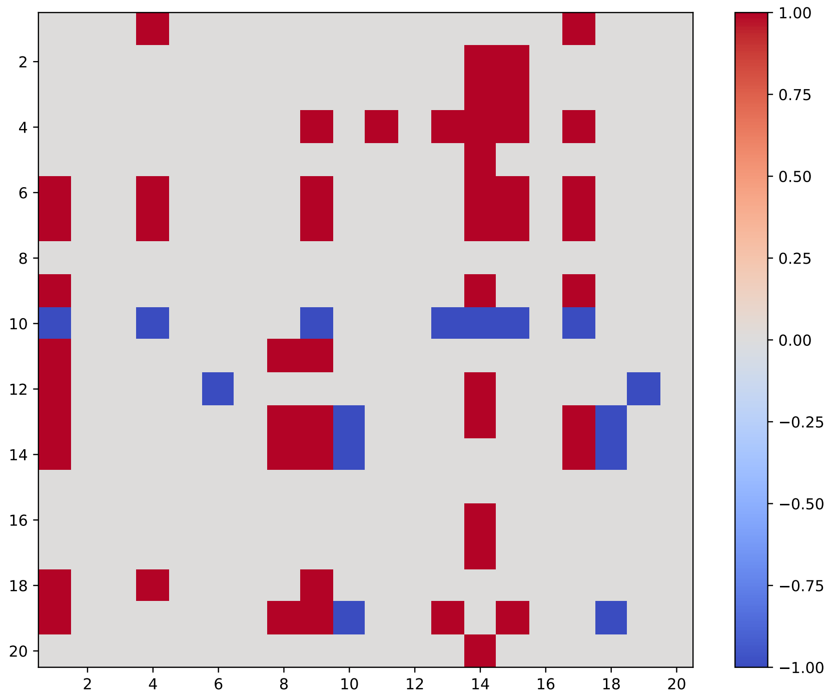

Figure S2: Heat map of the true GRN of THP-1 data set.

regulation and no regulation. Notice that almost all values are 0.

S5 Detailed performance measurements

References

Yue Wang, Peng Zheng, Yu-Chen Cheng, Zikun Wang, and Aleksandr Aravkin. Wendy: Covariance dynamics based gene regulatory network inference. *Mathematical Biosciences*, 377:109284, 2024.

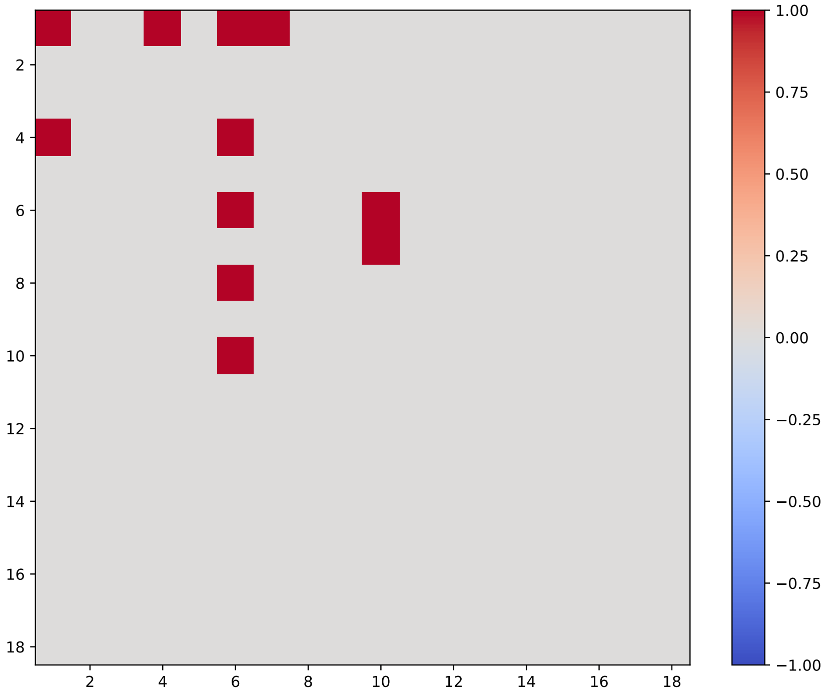

Figure S3: Heat map of the true GRN of hESC data set.

Method	$\sigma = 0.01$ AUROC	$\sigma = 0.01$ AUPRC	$\sigma = 0.1$ AUROC	$\sigma = 0.1$ AUPRC	$\sigma = 1$ AUROC	$\sigma = 1$ AUPRC	Average AUROC	Average AUPRC
WENDY	0.6290	0.5897	0.6654	0.6109	0.6008	0.5782	0.6317	0.5929
TRENDY	0.7533	0.6908	0.8547	0.7519	0.6678	0.6087	0.7586	0.6838
nWENDY	0.5227	0.5431	0.5131	0.5447	0.5045	0.5314	0.5134	0.5397
bWENDY	0.5227	0.5413	0.5129	0.5431	0.5052	0.5312	0.5136	0.5385
GENIE3	0.4487	0.5378	0.4062	0.5061	0.3965	0.4881	0.4171	0.5107
tGENIE3	0.7844	0.7189	0.8703	0.7672	0.6757	0.6040	0.7768	0.6967
nGENIE3	0.4403	0.5241	0.3985	0.4976	0.3689	0.4748	0.4026	0.4988
bGENIE3	0.4418	0.5223	0.3983	0.4959	0.3693	0.4746	0.4031	0.4976
SINCERITIES	0.6493	0.5726	0.6783	0.5829	0.7154	0.5967	0.6810	0.5841
tSINCERITIES	0.7192	0.6185	0.7964	0.6637	0.7661	0.6294	0.7606	0.6372
nSINCERITIES	0.5401	0.5369	0.5394	0.5387	0.5332	0.5363	0.5376	0.5373
bSINCERITIES	0.5422	0.5375	0.5450	0.5409	0.5374	0.5381	0.5415	0.5388
NonlinearODEs	0.5026	0.5243	0.5076	0.5313	0.5068	0.5301	0.5057	0.5286
tNonlinearODEs	0.5487	0.5460	0.5976	0.5658	0.5162	0.5333	0.5542	0.5484
nNonlinearODEs	0.4971	0.5205	0.5053	0.5251	0.5073	0.5236	0.5032	0.5231
bNonlinearODEs	0.4972	0.5202	0.5058	0.5260	0.5081	0.5248	0.5037	0.5237

Table S1: AUROC and AUPRC scores of all 16 methods on SINC data set. We list the scores separately for data with $\sigma = 0.01$, $\sigma = 0.1$, $\sigma = 1$, and the average scores.

Method	DREAM4 AUROC	DREAM4 AUPRC	THP-1 AUROC	THP-1 AUPRC	hESC AUROC	hESC AUPRC
WENDY	0.4899	0.2080	0.5261	0.3972	0.4997	0.0392
TRENDY	0.5341	0.2177	0.5557	0.3669	0.5311	0.0376
nWENDY	0.5417	0.2254	0.6112	0.4203	0.4971	0.0372
bWENDY	0.5421	0.2231	0.6106	0.4205	0.5070	0.0402
GENIE3	0.5636	0.2286	0.4484	0.3546	0.5913	0.0468
tGENIE3	0.4589	0.1799	0.5506	0.3781	0.6008	0.0435
nGENIE3	0.5632	0.2261	0.4861	0.3642	0.5744	0.0462
bGENIE3	0.5741	0.2284	0.4792	0.3623	0.5767	0.0488
SINCERITIES	0.4908	0.1919	0.6261	0.3852	0.4198	0.0261
tSINCERITIES	0.4995	0.2034	0.5251	0.3412	0.4871	0.0294
nSINCERITIES	0.4999	0.1856	0.5956	0.3900	0.1955	0.0199
bSINCERITIES	0.5040	0.1846	0.6067	0.3798	0.1842	0.0196
NonlinearODEs	0.4806	0.1705	0.5338	0.3486	0.5971	0.0534
tNonlinearODEs	0.5712	0.2452	0.4808	0.3302	0.6233	0.0641
nNonlinearODEs	0.4791	0.1772	0.5521	0.3482	0.6008	0.0466
bNonlinearODEs	0.4856	0.1666	0.5544	0.3498	0.6040	0.0633

Table S2: AUROC and AUPRC scores of all 16 methods on DREAM4, THP-1, and hESC data sets.
